# Diving of Great Shearwaters (*Puffinus gravis*) in Cold and Warm Water Regions of the South Atlantic Ocean

**DOI:** 10.1371/journal.pone.0015508

**Published:** 2010-11-30

**Authors:** Robert A. Ronconi, Peter G. Ryan, Yan Ropert-Coudert

**Affiliations:** 1 Department of Biology, Dalhousie University, Halifax, Canada; 2 Percy FitzPatrick Institute of African Ornithology, DST/NRF Centre of Excellence, University of Cape Town, Rondebosch, South Africa; 3 Institut Pluridisciplinaire Hubert Curien, Departement Ecologie, Physiologie and Ethologie, Strasbourg, France; Institute of Ecology, Germany

## Abstract

**Background:**

Among the most widespread seabirds in the world, shearwaters of the genus *Puffinus* are also some of the deepest diving members of the Procellariiformes. Maximum diving depths are known for several *Puffinus* species, but dive depths or diving behaviour have never been recorded for great shearwaters (*P. gravis*), the largest member of this genus. This study reports the first high sampling rate (2 s) of depth and diving behaviour for *Puffinus* shearwaters.

**Methodology/Principal Findings:**

Time-depth recorders (TDRs) were deployed on two female great shearwaters nesting on Inaccessible Island in the South Atlantic Ocean, recording 10 consecutive days of diving activity. Remote sensing imagery and movement patterns of 8 males tracked by satellite telemetry over the same period were used to identify probable foraging areas used by TDR-equipped females. The deepest and longest dive was to 18.9 m and lasted 40 s, but most (>50%) dives were <2 m deep. Diving was most frequent near dawn and dusk, with <0.5% of dives occurring at night. The two individuals foraged in contrasting oceanographic conditions, one in cold (8 to 10°C) water of the Sub-Antarctic Front, likely 1000 km south of the breeding colony, and the other in warmer (10 to 16°C) water of the Sub-tropical Frontal Zone, at the same latitude as the colony, possibly on the Patagonian Shelf, 4000 km away. The cold water bird spent fewer days commuting, conducted four times as many dives as the warm water bird, dived deeper on average, and had a greater proportion of bottom time during dives.

**Conclusions/Significance:**

General patterns of diving activity were consistent with those of other shearwaters foraging in cold and warm water habitats. Great shearwaters are likely adapted to forage in a wide range of oceanographic conditions, foraging mostly with shallow dives but capable of deep diving.

## Introduction

Shearwaters (*Puffinus* spp.) are among the most widespread seabirds throughout much of the world's oceans, adapted for foot and wing-propelled diving [1], [2], [3], with several species undertaking trans-equatorial migrations [4], [5]. Although many procellariiforms perform shallow (1–5 m) dives to capture prey [6], [7], studies using depth recorders have revealed the relatively deep (>50 m) diving abilities of *Puffinus* shearwaters. These shearwaters exhibit maximum diving depths that are allometrically scaled and comparable in depth to those of penguins and alcids [2]. However, maximum diving depths have been recorded for only 5 of the 20 or so species of *Puffinus* using capillary depth gauges [2], [8], [9], [10]. More recently, dives of sooty shearwater (*P. griseus*) have been recorded using archival tags sampling every 24 to 32 seconds [11], [12]. The use of high sampling rate time-depth-recorders (TDRs), which trace individual dives and record time spent at various depths, have been used on only one Balearic shearwater (*P. mauretanicus*) sampling depth every 4 seconds [13]. Thus, very little is known about the diving ecology of this genus.

We report the first deployments of TDRs on great shearwaters (*P. gravis*), sampling depth and temperature during foraging trips in the early incubation period. The great shearwater is the largest *Puffinus* [14] and nests almost exclusively on islands in the Tristan da Cunha group and Gough Island in the central South Atlantic Ocean [15], [16]. While breeding, they occupy a wide range of marine habitats from Polar Frontal zones to neritic waters of coastal South America and Africa [17]. Ocean temperatures, recorded during dives, were compared to remote sensing data and movement patterns of other shearwaters tracked by satellite telemetry, in order to estimate the likely foraging ranges of the TDR-equipped birds. The high sampling rate (2 s) used in our study allowed us to investigate of maximum dive depths, dive frequency and dive profiles in relation to diel cycles while foraging in two contrasting oceanographic regions.

## Methods

### Ethics Statement

Access to the study colony and work on shearwaters was granted full permission by the Tristan da Cunha government. While the Tristan government does not require animal ethics approval to conduct work on animals, we adopted tagging protocols used by R.A.R. on great shearwaters in Canadian waters and approved by the Institutional Animal Use and Care Committee, University of North Carolina Wilmington (certificate #2005-003 and #2007-007). Banding permits were issued by the Canadian Wildlife Service (permit #10480 S) with special permission granted to band birds outside of North America.

### Data collection and analyses

**Figure 1:**
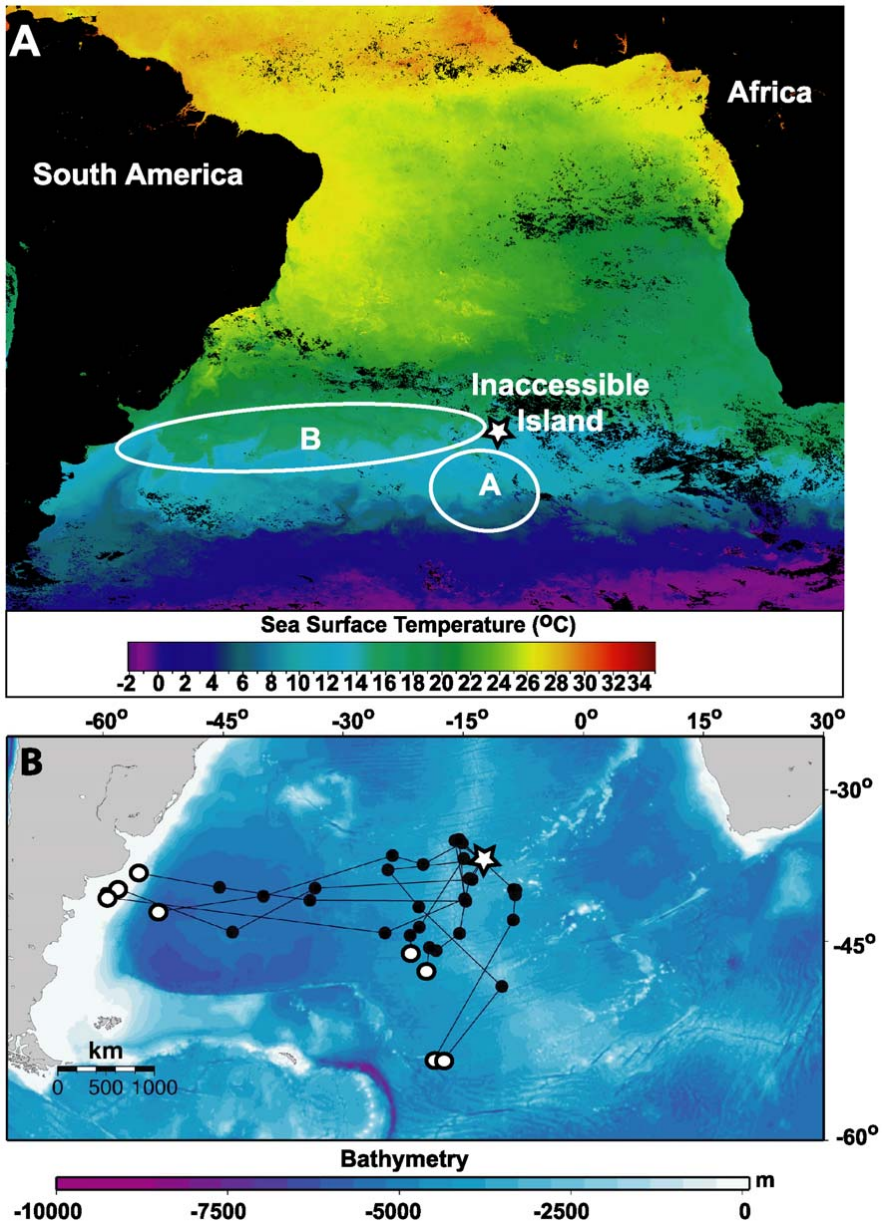
Sea-surface temperature and foraging ranges of great shearwaters during first post-laying foraging trips. A. Sea-surface temperature map (8-day averaged Modis Aqua data) during the TDR deployment period, 9–16 November, 2009. Ellipses indicate probable foraging ranges, based on TDR temperature profiles (Table 3), of birds A and B. B. Movements of 8 male Great Shearwaters tracked by satellite telemetry during the first nine days following their initial incubation shift on Inaccessible Island. Tags were deployed on 21 November, 2009, and departure dates ranged from 22 Nov to 07 Dec. Duty cycled tags transmitted every second day, and dots represents averaged daily locations for odd numbered days one through seven (•) and on day nine (○).

Breeding adult great shearwaters were accessed from nesting burrows on Inaccessible Island (37°18′S, 12°40′W; Fig. 1), which supports an estimated breeding population of ∼2 million pairs [18]. Devices were deployed on four female shearwaters on 7 November 2009 within one day of egg laying to record the diving behaviour during the first post-laying foraging trip. TDRs (G5; Cefas Technology Limited, UK) were attached to the central tail feathers with waterproof Tesa tape and two plastic cable-ties. Each device weighed ∼5 g (4 g device and ∼1 g of attachment materials), roughly 0.6% of the shearwaters' body mass at the time of deployment. To maximize data collection with the data storage capacity of the device, TDRs were programmed to begin recording at 05:00 GMT (local time) on the day following attachment, and continuously recorded pressure (depth) every 2 sec and temperature every 3 sec, yielding ∼10 d of data. The resolution of the depth sensor was <0.04 m and the absolute temperature accuracy of the thermistor was ±0.1°C. Burrows were checked every second day until females returned from their foraging trip. Devices were recovered from three individuals, but files were corrupted on one, so data were only available from two birds. The final bird had not returned to relieve its partner when we left the island, 25 days after deployment. In total, 48 burrows were monitored (4 study, 44 control) of which 22 (45%) failed due to predation [19] or abandonment, and in 12 of the 44 control burrows (27%) females had also not returned by the final nest check, 1 Dec 2009 (17 to 23 days after laying).

The TDR deployments occurred simultaneously during a larger study using satellite telemetry (PTT - Platform Terminal Transmitters) to track the movements of great shearwaters. A subset of these data was used to provide context for the likely foraging ranges of TDR-equipped birds. Eight PTTs (32 g Kiwisat 101, Sirtrack, Havelock North, NZ; ∼3–4% of shearwater body mass) were deployed on male great shearwaters, 21 Nov 2009, during their first incubation shift. Tags were duty cycled to turn on for 6 hrs at 10:00 UTC every second day. Foraging ranges were evaluated from the first 9 days once each male had departed the colony after their first incubation shift, which provides the most comparable data to the 10-d TDR recordings. Sea-surface temperature (SST) data were integrated with tracking data using Satellite Tracking and Analysis Tools (STAT [20]). Using positions with location class ≥0 (estimated accuracy typically within 4 km [21]), we calculated a single averaged location and SST for each day for each bird. Distance of each location to the colony was measured using ArcGIS 9.3 (ESRI, Redlands, CA, U.S.A.).

Downloaded TDR data were analyzed using IGOR Pro (Wavemetrics Inc., USA, 2008, Version 6.04). Data were corrected for surface drift and a number of parameters were automatically extracted for each dive: time of start and end of dives, maximum dive depth (m), total dive and post-dive duration (s), descent, bottom and ascent phases duration (s), and the number of undulations in the dive profiles (s). A dive started and ended when consecutive depth readings became greater and smaller than 0.5 m, respectively. The start and end of bottom phases were defined as the first and last time in a dive when the rate of change of depth became <0.25 m.s^−1^ [22].

Sunrise and sunset times (civil dawn/dusk) were used from two locations to evaluate diel patterns in foraging: Inaccessible Island and the Patagonia shelf region (37°S, 55°W). All breeding great shearwaters tracked by satellite tags from Inaccessible Island during the same period foraged between these two areas (Fig. 1). We estimated the likely general foraging locations by comparing temperature data recorded by the TDRs (using the minimum temperature measured within a few minutes of a dive) with sea surface temperature (SST) maps during the deployment period (Fig. 1; 8-day binned map from 9–16 Nov. 2009; Modis Aqua, http://oceancolor.gsfc.nasa.gov/ftp.html). Oceanographic zones were broadly defined focusing on surface temperatures in the central South Atlantic region [23]: Polar Front (2–4°C), Sub-Antarctic Frontal Zone (5–9°C), and Sub-tropical Frontal Zone (10–17°C).

All statistics were analyzed with SPSS 15.0. T-tests were used to compare dive parameters between individuals and Analysis of Variance (ANOVA) was used to compare dive and temperature parameters among days for individual birds.

## Results

Tags were recovered from two birds after 17 d (bird A) and 22 d (bird B) foraging trips. Pre- and post-trip masses were 770/900 g and 810/975 g for birds A and B, respectively, averaging a daily mass gain of 7.5 and 7.6 g⋅d^−1^. Non-instrumented females, for which departure and return dates were recorded (n = 10), had mean trip durations of 18.8 d (range 16–22), mean post-trip mass of 929 g (845–975), and average daily mass gains of 8.7 g⋅d^−1^ (6.1–13.8). During the first 10 days of foraging, we recorded a total of 930 dives, with bird A performing nearly four times as many dives (n = 739) as bird B (n = 191). The deepest dive was to 18.9 m, and this was also the longest dive, lasting 40 s. It was made by bird A, which also exhibited greater average maximum dive depths (t_989_ = 2.53, p = 0.012) and durations (t_989_ = 3.01, p = 0.003) than bird B (Table 1). Most dives were shallow (>50% of dives were <2 m deep for both individuals; Table 1), but bird A performed many more dives deeper than 10 m (nearly 10%), compared to bird B (which only dived >10 m once; max. 10.9 m).

**Table 1:** Diving behaviour of two female Great Shearwaters during 10-day Time-depth Recorder deployments.

		Dive Duration (s)		Dive Depth (m)		% of Dives			
Bird	n	Max	Mean (SD)	Max	Mean (SD)	<2 m	2–5 m	5–10 m	>10 m
A	739	40	7.9 (8.5)	18.9	3.3 (3.8)	58.1	21.6	10.8	9.5
B	191	22	6.0 (4.2)	10.9	2.6 (2.0)	51.8	34.6	13.1	0.5

The measurement of bottom phase duration allowed for simple classification and comparison of dives which contained a bottom phase (BP) and those that did not (hereafter V-shaped dives; Table 2). Most dives (67.7% overall) were V-shaped and significantly shallower than BP dives for both bird A (2.0 vs. 5.8 m; t_737_ = 14.9, p<0.001) and bird B (2.4 vs. 3.3 m; t_189_ = 2.81, p = 0.005). During BP dives, both birds spent a similar proportion of the dive time in the bottom phase (42 and 39%), but the overall dive duration and maximum depth was greater for bird A (t_301_>3.69, p<0.001). This suggests that bird A spent more time pursuing prey at depth; the longest bottom phase was 24 s of a 28 s dive. During BP dives, there were no significant differences between individuals in either decent rates (t_301_ = 0.67, p = 0.505) or ascent rates (t_301_ = 1.09, p = 0.277) which averaged 1.14 and 1.35 m⋅s^−1^ respectively (Table 2).

**Table 2:** Characteristics of V-shaped dives and bottom-phase dives by two female great shearwaters.

		V-shaped Dives		Dives with Bottom Phase (BP)				
Bird	n	% of dives	Mean depth (m)	BP Duration (s)	% of dive time in BP	Mean depth (m)	Descent rate (m.s^−1^)	Ascent rate (m.s^−1^)
A	739	65.5	2.0±2.3	6.0±4.5	42.1	5.8±4.8	1.15±0.48	1.37±0.69
B	191	74.9	2.4±1.9	3.4±1.9	39.2	3.3±1.9	1.10±0.53	1.26±0.60
**Combined**	**930**	**67.4**	**2.1±2.2**	**5.6±4.1**	**41.6**	**5.4±4.6**	**1.14±0.49**	**1.35±0.68**

Diving behaviour showed considerable variability among days. Dive frequency varied significantly among days for both individuals (*X^2^* = 194, df = 9, p<0.001; Table 3), with both birds making few dives on day 1, suggesting that they were either resting near the colony or commuting to foraging areas. Bird B had two further days with no or few (3) dives, suggesting a longer commuting time by this individual. Mean dive depth and SST associated with each dive varied significantly among days for both birds (ANOVA: F>6.96, n = 10 days, p<0.001 for all comparisons; Table 3), suggesting that birds moved among habitats, diving differently within each. Bird A foraged primarily in the northern limits of the Sub-Antarctic Frontal Zone (8 to 10°C; hereafter “cold” water) whereas bird B exploited a wider range of warmer water temperatures (10 to 16°C) characteristic of the Sub-tropical Frontal Zone. Inspection of SST maps during this period (Fig. 1) suggests that bird A (cold water) foraged in a band of water at 42–48° S that stretched from South Africa to the Patagonian Shelf. At its closest, this region was approximately 800 km SSW of Inaccessible Island. The warmer water in which bird B foraged mainly occurred between 36–40° S, also stretching across the South Atlantic Ocean and overlapping with Inaccessible Island. Average SST within a 4 km radius of Inaccessible was 12.7±1.4°C (SD), nearly equal to that of bird B on day 1.

**Table 3:** Diving behaviour of two female great shearwaters during the first 10 days of the first foraging trip post egg-laying.

	Bird A				Bird B			
Day	# dives	mean SST	mean depth	max depth	# dives	mean SST	mean depth	max depth
08-Nov	6	10.4	0.9	2.1	7	12.6	1.1	2.3
09-Nov	30	10.3	9.2	18.9	0	*		
10-Nov	47	10.1	4.3	12.1	3	16.0	1.2	1.9
11-Nov	61	10.0	4.5	17.7	32	13.3	2.9	7.9
12-Nov	31	10.3	2.1	12.5	16	15.7	1.7	6.0
13-Nov	44	9.1	3.9	16.7	12	10.0	3.0	10.9
14-Nov	120	8.7	2.6	16.9	0	*		
15-Nov	120	8.2	2.4	14.5	30	13.4	4.0	8.2
16-Nov	124	8.2	3.0	17.1	25	13.4	3.4	7.2
17-Nov	156	8.4	3.1	16.3	66	13.2	1.8	7.1
**Mean ± SD**	**74±51**			**14.5±4.9**	**19±20**			**6.4±3.0**

Sea-surface temperature (SST) was recorded by the logger (see text).

*no dives recorded, presumably in transit.

Male shearwaters tracked by satellite telemetry illustrate a dual foraging strategy where half of the individuals traveled south towards the Sub-Antarctic Front, and the others traveled west to the Patagonian Shelf (Fig. 1). Males departed the colony between 21 Nov and 07 Dec, which overlaps with the foraging trips of females but not with the 10-d TDR measurements. Within nine days of departing the colony, southward bound birds had traveled between 1200 and 1950 km and westward bound birds traveled ∼4000 km to within 200 km of the South American coast (Table 4; Fig. 1). Sea-surface temperatures associated with their locations (Table 4) showed that southward and westward bound birds foraged in cool (min. temp. typically <10°C) and warm waters (min. temp. >10°C) respectively, corroborating the patterns inferred from TDR data and SST maps. By day nine, two south bound individuals had reached the Polar Front (2.2°C; Table 4).

**Table 4:** Sea surface temperature (SST) and distance traveled by 8 male great shearwaters tracked by satellite telemetry.

	Westward bound birds (n = 4)			Southward bound birds (n = 4)		
Day	SST (min)	SST (max)	Mean distance from colony (km)	SST (min)	SST (max)	Mean distance from colony (km)
1	10.4	16.5	311	11.3	14.8	183
3	14.2	15.5	853	8.9	17.8	633
5	10.1	18.0	1723	9.5	13.7	849
7	13.5	16.5	3288	9.6	13.6	1048
9	13.3	15.1	3813	2.2	8.5	1600*

Data are reported every second day during the first 9 days following the initial incubation shift. SST data were integrated with tracking data using Satellite Tracking and Analysis Tools (STAT [20]) which associated locations with most recent remote sensing imagery.

*two birds at ∼1250 km and two at ∼1950 km.

**Figure 2:**
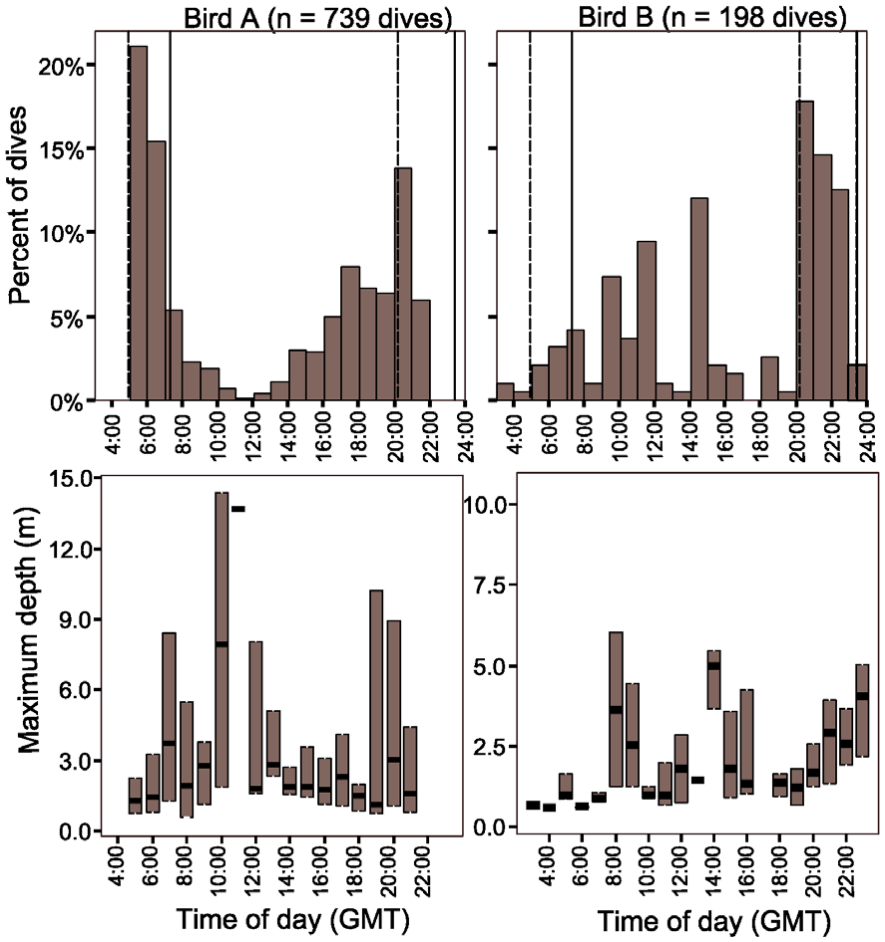
Diel patterns in diving frequency and depth by two female great shearwaters. Vertical lines reference sunrise and sunset times (civil dawn/dusk) for Inaccessible Island (dashed lines) and the Patagonia Shelf region (solid lines) from 20 November 2009. Box plots (lower graphs) indicate median dive depth and first and third quartiles (boxes presented only for hours with n>2 dives).

Nearly all dives occurred during daylight hours (Fig. 2), with a peak at dusk (both birds) and dawn (bird A). Assuming that bird A remained south of Inaccessible at approximately the same longitude, 35.6 and 28.3% of dives occurred within 2 h of sunrise and sunset, respectively. Assuming that bird B foraged as far away as the Patagonian Shelf, 17.3 and 16.8% of dives occurred within 2 h of sunrise and sunset, respectively, with 42.4% of all dives within 3 h of sunset. Without knowing the precise sunrise/set times at each diving location, we can only be certain that 3/930 dives (<0.5%) occurred outside of daylight hours from either region. Dive depth varied significantly among daylight hours (ANOVA bird A F_15,722_ = 7.56, p<0.001; bird B F_17,171_ = 4.25, p<0.001), but no clear diel pattern was apparent (Fig. 1).

## Discussion

This study reports the first measurements of diving behaviour for great shearwaters and is only the second to use TDRs on a *Puffinus* shearwater. The maximum recorded depth (18.9 m) was less than has been recorded for other species: Balearic shearwater (26 m, n = 1 bird [13]), Audubon's shearwater *P. lherminieri* (35.4 m [2]), black-vented shearwater *P. opisthomelas* (52 m [9]), wedge-tailed shearwater *P. pacificus* (66.4 m [2]), sooty shearwater (67–70 m [10], [12]), and short-tailed shearwater *P. tenurostris* (70.6 m [24]). Although TDR data from the present study clearly demonstrate that great shearwaters frequently dive deeper than previously thought [1], they did not dive as deeply as might be expected by allometric relationships between mass and maximum diving depth [2], given that the great shearwater is the largest member of the genus [14]. Similar sized shearwaters of the genus *Calonectris* typically perform comparatively shallow dives (mean 2.7 m, max 5.5 m [6]), though this large winged genus is adapted for gliding in tropical waters [25]. Shallow diving was also common for great shearwaters in the Sub-tropical and Sub-Antarctic Frontal Zones (>50% of dives <2 m, this study). The maximum diving depths reported in this study are likely underestimates of their true maximum potential as we only recorded diving by two individuals. Nevertheless, the mean daily maximum dive depth of bird A (14.5 m; Table 3) is comparable to the mean maximum dive depths for several other *Puffinus* species [2], thus, the general patterns reported may be representative of the diving behaviour of great shearwaters. The fact that the birds put on weight during their foraging trips and returned after a similar period to other, control birds, indicates that the TDRs had little impact on their performance.

Although most shearwaters are well adapted for underwater swimming [1], [25], virtually nothing is know of the dive profiles for this genus. Most dives by one Balearic shearwater were V-shaped [13], although 4 s recording interval used in that study may have lacked the precision to detect short bottom phases. By sampling depth twice as frequently as [13], we were better able to examine dive profiles of great shearwaters. Although most dives were V-shaped, dives with a bottom phase by bird A had an average bottom phase lasting 6.0 s (max 24 s) which accounted for approximately 42% of the total dive time. This suggests that this shearwater was pursuing prey at depth. By comparison, bird B made fewer dives with a bottom phase. Its relatively few, short-duration dives are typical of birds scavenging for offal and discards [26], possibly at fishing vessels operating on the Patagonian Shelf [27].

The average bottom time and descent velocity were less than those of other foot and wing-propelled diving seabirds (great cormorants *Phalacrocorax carbo* and little penguins *Eudyptula minor*) that dive to similar depths [22], which would be expected since these birds are shaped for flight rather than underwater locomotion, and also since they dive in the upper few metres of the water column where buoyancy is greatest [28]. However, the ability of shearwaters to descend the water column deserves further investigation, especially regarding the speed and angle adopted during dives to different depths, which will require instruments such as accelerometers sampling at even faster rates [29].

Several species of procellarids are thought to commonly forage at night [30], [31], but we found little evidence of nocturnal diving. Great shearwaters consume large amounts of squids [32], [33], [34], but where and when they obtain this prey is unknown and our data suggest little to no nocturnal diving by this species, as has been reported for other *Puffinus* shearwaters [12], [13]. Instead, great shearwaters showed increased diving frequency around dawn and dusk - similar to patterns reported for sooty shearwaters [11]. No clear diel patterns in dive depth were apparent, although diving depths appear to have been greatest in mid morning and again before sunset, which shows partial overlap with deeper diving periods for sooty shearwaters [11]. Great shearwater diet during breeding periods is poorly known, with the few chicks and adults sampled containing mainly squid (flesh and beaks) and occasionally fish and crustaceans [32], [35]. It is possible that these prey are obtained during shallow dives near dawn and dusk when vertically migrating prey may be closer to the ocean surface [36] or during surface foraging at night.

The habitats used by the two birds in this study differed with bird A foraging consistently in cool (8–10°C) and bird B in more variable, warmer (10–16°C) waters, characteristic of Sub-Antarctic and Sub-tropical Frontal Zones [23] respectively. Breeding short-tailed and sooty shearwaters from New Zealand and Australia are known to undertake both short (<500 km) foraging trips within warm neritic waters (13–17°C) and long (∼2000 km) trips to cold polar frontal zones (4–7°C) [11], [12], [24]. The temperatures recorded by TDRs correspond with locations of male great shearwater tracked by satellite tags during the first 9 d after departing Inaccessible Island. Males in 8–10°C water were approximately 600–1200 km SSW of Inaccessible, whereas those in 13–16°C water ranged up to 4000 km west towards the Patagonian Shelf which was reached within 7 days by one individual. Thus it seems probable that bird B foraged as far away as the Patagonian Shelf whereas bird A foraged in pelagic waters in the northern Sub-Antarctic Frontal Zone. We observed large differences in diving behaviour between these regions, with more commuting days (due to greater distance traveled), shallower dive depths, and fewer dives in warmer waters. This may have been due to differences between individual diving abilities rather than habitat associations, but the deepest dives of bird B (10.9 m) also occurred in the coldest water (10°C) occupied by this bird. Likewise, sooty shearwaters demonstrated greater diving effort (including more trips, more dives and deeper dives) in cold water regions along the Polar Front [11], [12], mirroring patterns observed in this study. Without information on prey depth or abundance, it is not possible to speculate on the foraging efficiency of great shearwaters diving in warm and cold waters during this study. Nevertheless, both birds returned to the colony with replenished body reserves, suggesting that both long-distance trips to warm water regions and short-distance trips to Sub-Antarctic Frontal Zones, may provide profitable foraging strategies for this species.
